# Supplementary material for: Lorentz microscopy of optical fields
Source: Nat Commun. 2023 Oct 17;14:6545. doi: 10.1038/s41467-023-42054-3 (PMC10582189; doi:10.1038/s41467-023-42054-3)
Supplement: Supplementary file 1 — Supplementary Information [file 41467_2023_42054_MOESM1_ESM.pdf]

Lorentz Microscopy of Optical Fields

John H. Gaida,[1,2] Hugo Lourenço-Martins,[1,2] Sergey V. Yalunin,[1,2] Armin Feist,[1,2] Murat Sivis,[1,2] Thorsten Hohage,[3] F. Javier García de Abajo,[4,5] and Claus Ropers[1,2]

[1]*Max Planck Institute for Multidisciplinary Sciences, 37077 Göttingen, Germany*
[2]*4th Physical Institute – Solids and Nanostructures, University of Göttingen, 37077 Göttingen, Germany*
[3]*Institute of Numerical and Applied Mathematics, University of Göttingen, 37083 Göttingen, Germany*
[4]*ICFO-Institut de Ciencies Fotoniques, The Barcelona Institute of Science and Technology, 308860 Castelldefels (Barcelona), Spain*
[5]*ICREA-Institució Catalana de Recerca i Estudis Avançats, 08010 Barcelona, Spain*

Contents

Supplementary Figure S1: Defocus series of the nanotip without inelastic electron light scattering. 2

Supplementary Figure S2: Polarization control of the optical near field. 3

Supplementary Figure S3: Simulated image contrast formation. 4

Supplementary Figure S4: Contribution of each plasmonic mode to the magnitude of the interaction coefficient g. 5

Supplementary Figure S5: Reconstruction of simulated data. 6

Supplementary Figure S6: Phase contrast transfer function (PCTF) and point resolution. 6

Supplementary Figure S7: BEM simulation of different geometries. 7

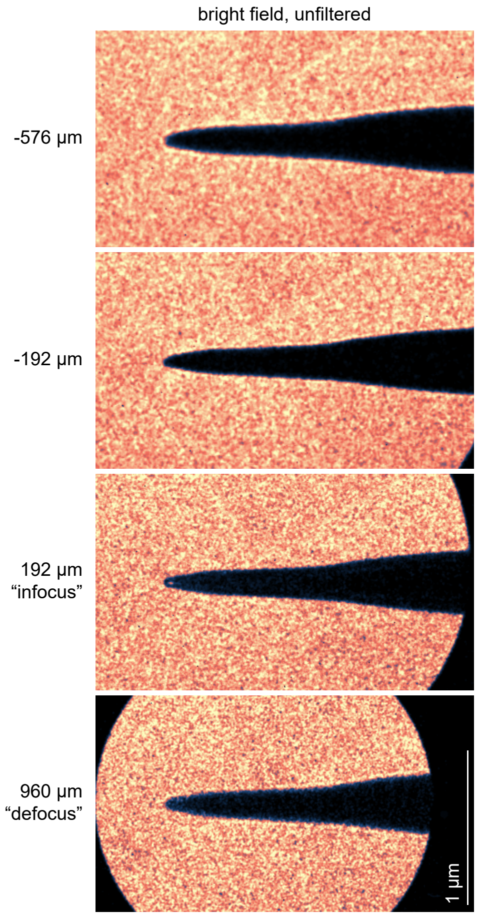

Supplementary Fig. S1 Defocus series of the nanotip without inelastic electron light scattering. A defocus series of unfiltered bright field images taken before time-zero at $-10\,\mathrm{ps}$ with otherwise identical microscopy settings including the illumination.

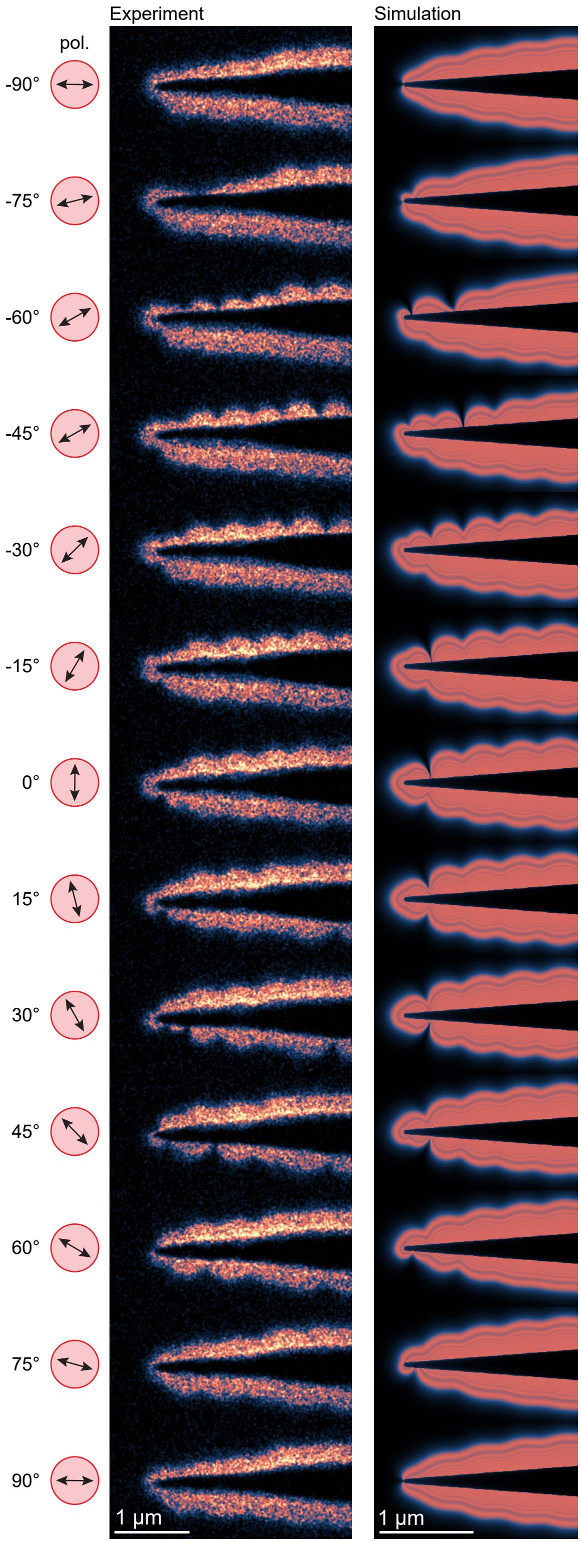

Supplementary Fig. S2 Polarization control of the optical near field. A controlled change of the linear polarization of the incident laser excitation changes the optical near field at the nanotip as imaged with energy-filtered TEM. Simulated images are shown for comparison showing similar behaviour.

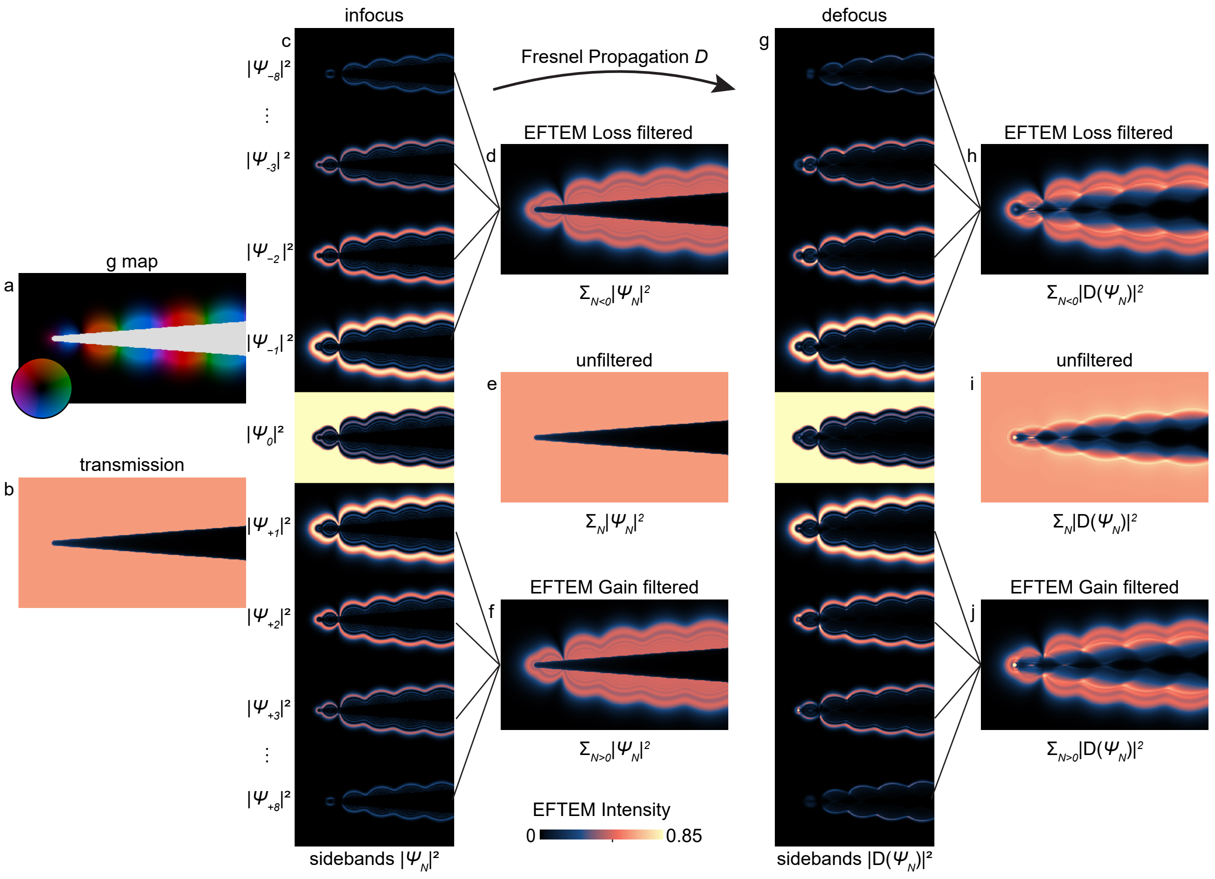

Supplementary Fig. S3 Simulated image contrast formation. From a given $g(x, y)$ (**a**) and transmission (**b**) maps of the specimen, the in-focus sideband magnitudes (**c**) can be calculated using Eq. 2.By summing up the loss (**d**), all (**e**) or the gain (**f**) sidebands, we can simulate the in-focus EFTEM images. Propagating the individual sidebands with the Fresnel propagator results in the defocused image contrast (**g-j**).

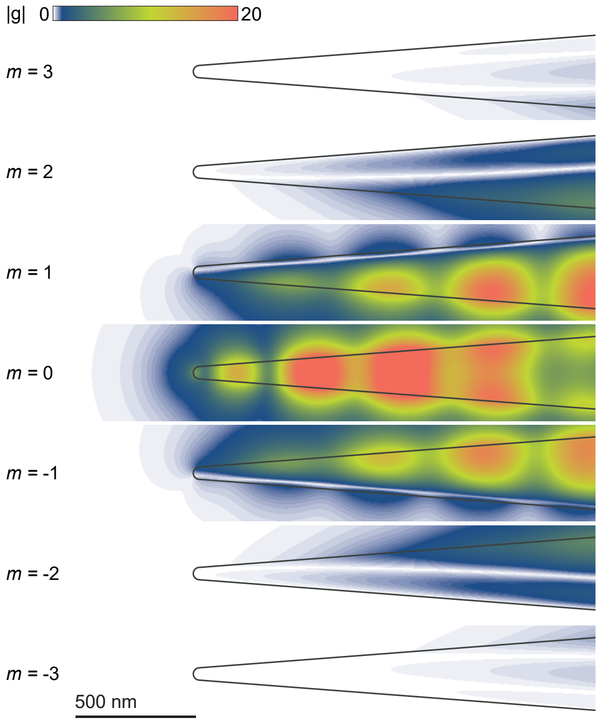

Supplementary Fig. S4 Contribution of each plasmonic mode to the magnitude of the interaction coefficient $|g|$. Due to the rotational symmetry of the tip, the coupling coefficient can be rigorously decomposed as a sum over azimuthal numbers m (i.e., $g = \sum_m g_m$). These plots show that the coupling coefficient is dominated by the $m = 0$ and $m = \pm 1$ components. Incidentally, the $m = \pm 1$ plots are asymmetric with respect to the tip axis because electron-beam positions in which the plasmons circulate along the same direction as the electron velocity are favoured.

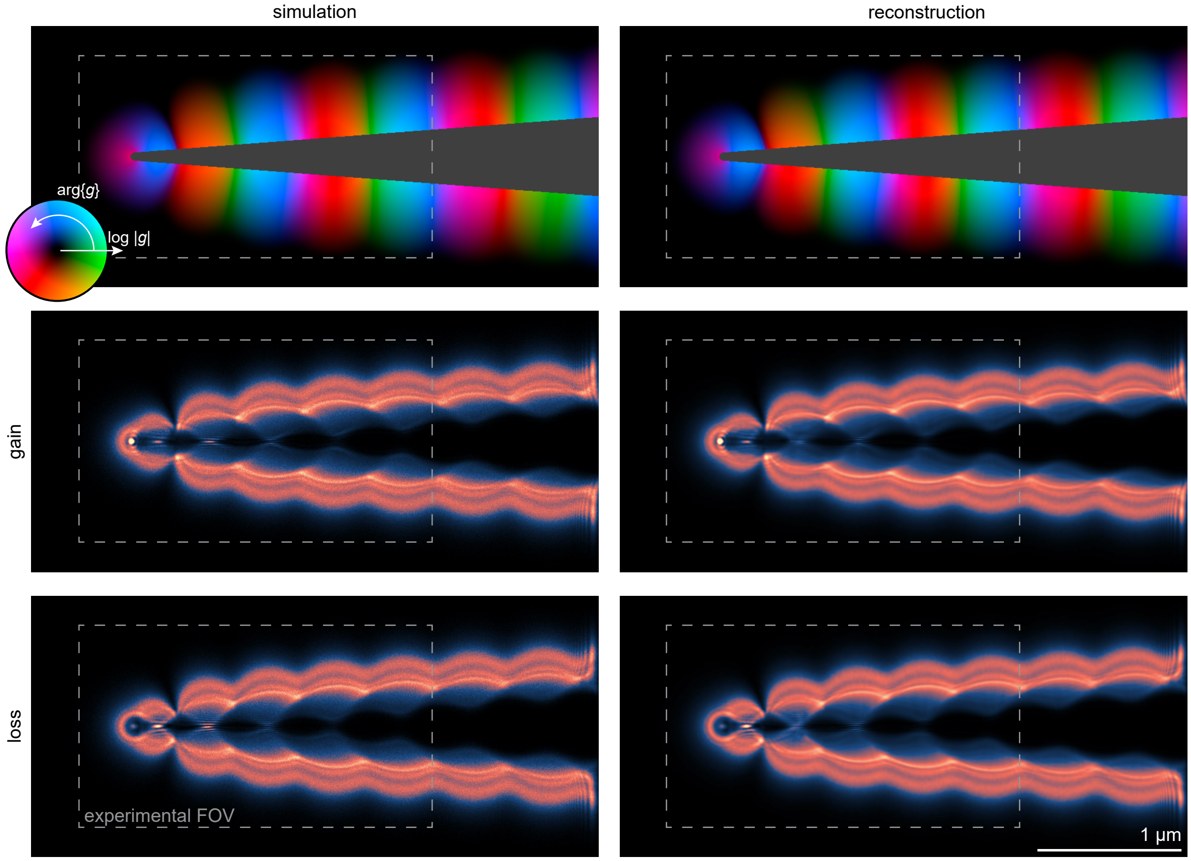

Supplementary Fig. S5 Reconstruction of simulated data. The g from the BEM simulation is used to calculate EFTEM micrographs for gain and loss, respectively. Experimental conditions are simulated by adding Poisson noise to the data following from a total number of electrons of 5 ů 10^7 that is comparable to the experimental conditions. The simulated micrographs are used to reconstruct $\arg\{g\}$ as described in the main text and the methods. The reconstructed phase approximates the BEM-simulated phase well up to minor deviations. The dashed line shows the experimental field of view in Fig. 3.

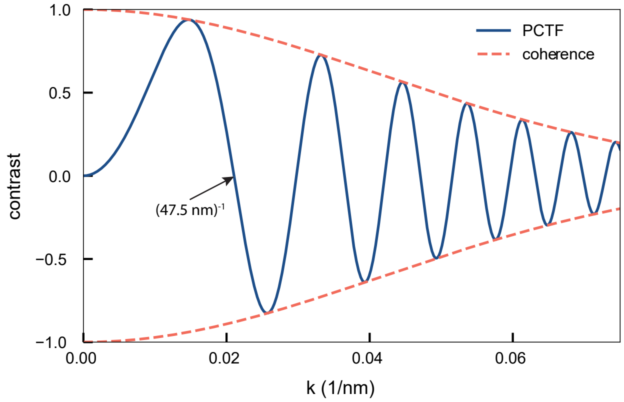

Supplementary Fig. S6 Phase contrast transfer function (PCTF) and point resolution. The PCTF in Lorentz TEM is dominated by the defocus and the coherence envelope, while spherical aberrations can be neglected at the typical Fresnel numbers (determined by defocus and FOV). A conservative indicator for the point resolution is given by the first zero crossing of the PCTF which corresponds to $(47.5\,\mathrm{nm})^{-1}$ for a defocus of 900 um as used in the experiments.

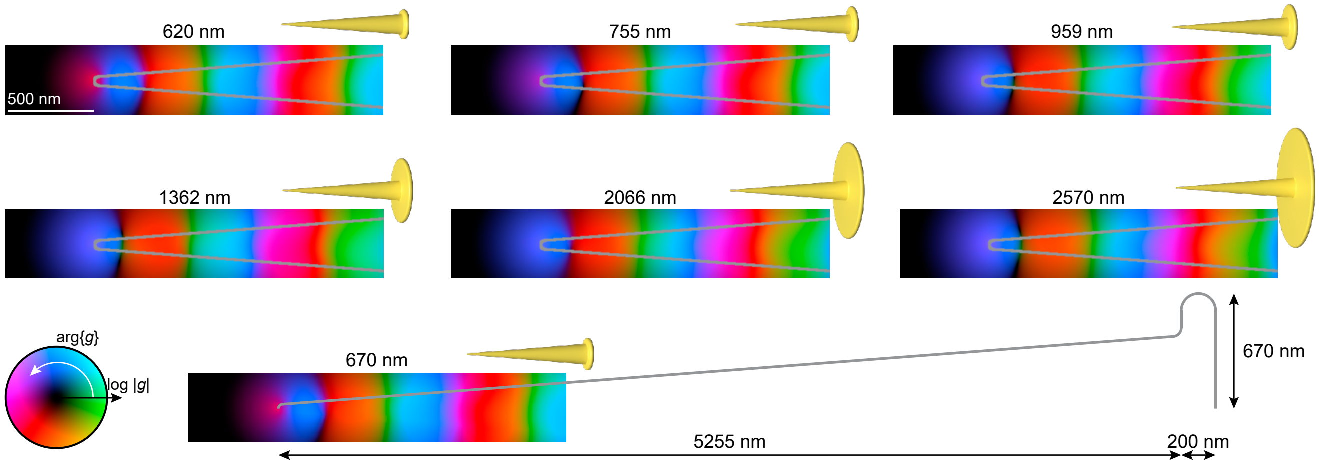

Supplementary Fig. S7 BEM simulation of different geometries. The size of the platform truncating the tip shaft geometry drastically changes the near field. The bottom row shows the complete contour to scale as used in the main text.
