# Supplementary figures and images for: Lorentz microscopy of optical fields
Source: Nat Commun. 2023 Oct 17;14:6545. doi: 10.1038/s41467-023-42054-3 (PMC10582189; doi:10.1038/s41467-023-42054-3)

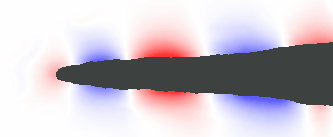

Supplement: Supplementary file 3 — Supplementary Movie S2 [file 41467_2023_42054_MOESM3_ESM.gif]
