# Supplementary material for: Lorentz microscopy of optical fields
Source: Nat Commun. 2023 Oct 17;14:6545. doi: 10.1038/s41467-023-42054-3 (PMC10582189; doi:10.1038/s41467-023-42054-3)
Supplement: Supplementary file 4 — Description of Additional Supplementary Files [file 41467_2023_42054_MOESM4_ESM.pdf]

Description of Additional Supplementary Files

Supplementary Movie S1: Gain Loss Lorentz PINEM The movie consists of alternating gain- and loss-filtered Lorentz PINEM images shown in Fig. 3 in the main text.

Supplementary Movie S2: Electric Field at Nanotip The movie shows the electric field at the nanotip, obtained from the reconstructed phase profile in Fig. 4 b in the main text.
